# Fabrication of ordered arrays of micro- and nanoscale features with control over their shape and size via templated solid-state dewetting

**DOI:** 10.1038/srep09823

**Published:** 2015-05-08

**Authors:** Jongpil Ye

**Affiliations:** 1 Department of Materials Science and Engineering, Inha University, Incheon 402-751, Korea

## Abstract

Templated solid-state dewetting of single-crystal films has been shown to be used to produce regular patterns of various shapes. However, the materials for which this patterning method is applicable, and the size range of the patterns produced are still limited. Here, it is shown that ordered arrays of micro- and nanoscale features can be produced with control over their shape and size via solid-state dewetting of patches patterned from single-crystal palladium and nickel films of different thicknesses and orientations. The shape and size characteristics of the patterns are found to be widely controllable with varying the shape, width, thickness, and orientation of the initial patches. The morphological evolution of the patches is also dependent on the film material, with different dewetting behaviors observed in palladium and nickel films. The mechanisms underlying the pattern formation are explained in terms of the influence on Rayleigh-like instability of the patch geometry and the surface energy anisotropy of the film material. This mechanistic understanding of pattern formation can be used to design patches for the precise fabrication of micro- and nanoscale structures with the desired shapes and feature sizes.

Dewetting of a thin film is a morphological evolution by which an initially continuous thin film evolves to form a discontinuous array of isolated islands. This phenomenon is driven by the minimization of energies associated with the film’s interfaces, and often occurs in the solid-state at elevated temperatures well below the film’s melting temperature. Solid-state dewetting of a thin film often begins with the formation of film edges by heterogeneous nucleation of holes that penetrate to the film-substrate interface. A large gradient in curvature at the film edges drives surface diffusion of atoms from the edges to the top of the film, causing the edges to retract and thicken^1^,^2^. The edges are later subject to a series of instabilities, including fingering instability^3^, pinch-off^4^,^5^, and Rayleigh-like instability^6^, eventually resulting in the formation of a discontinuous pattern of particles^1^.

Solid-state dewetting has recently been purposefully induced to produce patterns for various applications, notably in areas such as catalysis and plasmonics^7^,^8^,^9^. Unfortunately, patterns formed via solid-state dewetting of polycrystalline films are generally spatially disordered owing to a lack of symmetry at their surfaces and the stochastic nature of heterogeneous nucleation of holes. Hence, the development of methods for fabricating highly ordered patterns is indispensable to expand the applicability of this process. It has been shown that spatially ordered arrays of particles can be produced via dewetting of polycrystalline films deposited on topographically patterned substrates. The substrate topography modulates the curvature of the film and guides the evolution of the film surface during dewetting to form a particle array commensurate with it^10^. In the case of single-crystal films, they are crystallographically confined to evolve during dewetting into specific morphologies that are compatible with the symmetries of their surfaces. Accordingly, single-crystal films on flat substrates can be templated by pre-patterning them using simple planar photolithography, such that regular particle- or line-based structures are formed during dewetting. Thompson and I have recently demonstrated that the dewetting of patches patterned from 120 nm-thick single-crystal nickel films leads to the controllable formation of various regular patterns with smaller feature sizes and increased complexity. The shape characteristics of the dewetted patterns were explained in terms of a finite set of instabilities, including corner instability, pinch-off of edges, and Rayleigh-like instability, and the influences thereon of the surface energy anisotropy^11^. However, the previous study did not address the questions of a potential influence of the film material and thickness on the shape and size characteristics of dewetted patterns. These are important questions to be addressed prior to the generalization of this patterning method to a wider range of materials and scales. In the present study, I report dewetting results of patches patterned from single-crystal palladium and nickel films of different orientations and thicknesses. The patches dewet to form regular patterns of specific shapes that strongly depend on the width, orientation, thickness, and material of the patches. The geometric characteristics of the dewetted patterns show that the shape and scale of the dewetted features are controllable via the width, thickness, and orientation of the patches. The different dewetting behaviors of the palladium and nickel films are explained in terms of the surface energy anisotropies of the two materials, suggesting that templated-dewetting of single-crystal films can be used to pattern a wide range of materials of known surface energy anisotropy.

## Results and Discussion

### The effects of initial width and crystallographic alignment of patches on their evolution

**Figure 1:**
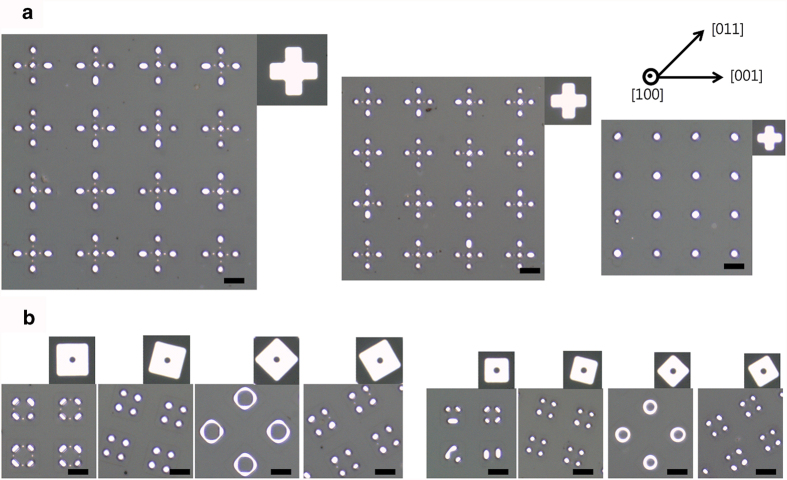
Dewetting of patches patterned from a 75 nm-thick Pd(100) film. (**a**) OM images of patterns produced via dewetting of cross patches. (**b**) OM images of patterns produced via dewetting of square patches with an internal circular hole. The initial patch is shown in the upper-right corner of the corresponding panel. The orientation of the film is indicated in the upper-right corner of the figure. The scale bars represent 5 μm.

Figure 1 shows the patterns formed during dewetting of patches patterned from a 75 nm-thick Pd(100) film. This film was annealed at 900 °C under 5 sccm of hydrogen and 200 sccm of argon for 1 h. As seen in the figure, the patches dewet to form particle- or line-based patterns of specific shapes depending on their initial shape, size, and in-plane alignment. The patterns shown in Fig. 1a were generated via dewetting of cross patches of varying arm widths. The arms were all initially aligned along the <001> directions. Cross patches with arm widths of 4.5 μm and 3.5 μm dewet to form five particles located at the centers of their bodies and arms (see the left two panels of (a)). Dewetting of these patches begins with edge retraction that occurs via surface diffusion driven by the curvature gradient at the patch edges. This edge retraction leads to the formation of patterns of lines narrower than in the initial patches. The overlap of diffusion fields at the convex corners of the patches causes more material to accumulate at the ends of the four arms than elsewhere^5^. This induces pearling at the ends of the line patterns, leading to the formation of surface perturbations. The perturbation subsequently grows to form isolated particles via Rayleigh-like instability. In contrast to the larger patches, the cross patches with arm widths of 2.5 μm evolve into a single particle (see the right-most panel of (a)). This implies that the wavelength of the surface perturbation in the smaller patterns is shorter than the critical wavelength for Rayleigh-like instability^6^. The in-plane orientation-dependent stability of patterns is clearly seen in Fig. 1b for the dewetting of square patches with an internal circular hole. As seen in the figure, patches with edges initially aligned normal to the <011> directions are more stable against Rayleigh-like instability than other patches. The surface energy of Pd is minimal at {111} orientations^12^, and facets of these orientations can be exposed only in thickened edges normal to the <011> directions in Pd(100) films. The critical wavelength for Rayleigh-like instability is substantially greater in lines that have these facets, leading to the greater stability of the patterns with edges normal to the <011> directions in Pd(100) films.

**Figure 2:**
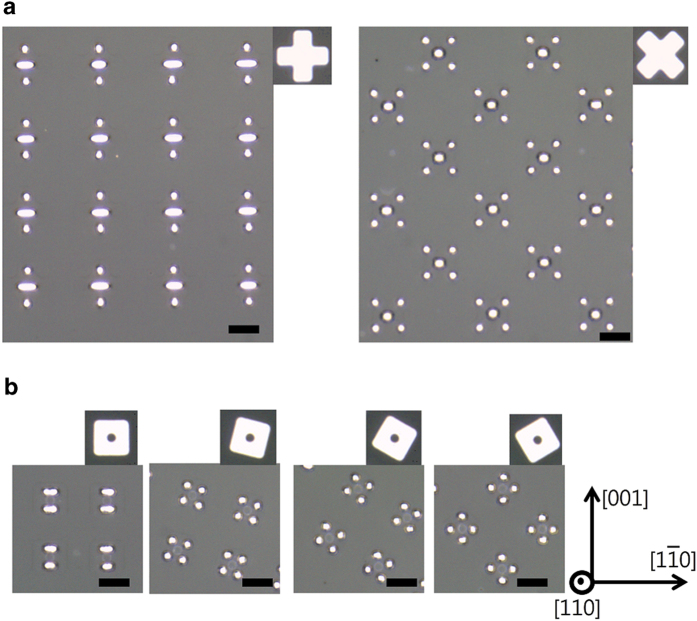
Dewetting of patches patterned from a 75 nm-thick Pd(110) film. (a) OM images of patterns produced via dewetting of cross patches. (**b**) OM images of patterns produced via dewetting of square patches with an internal circular hole. The initial patch is shown in the upper-right corner of the corresponding panel. The orientation of the film is indicated in the bottom-right corner of the figure. The scale bars represent 5 μm.

Results of dewetting for patches patterned from 75 nm-thick Pd(110) films are consistent with those shown in Fig. 1 in terms of the dependence of the line stability of the dewetted patterns on the crystallographic alignment of the patches. These films were annealed at 900 °C under 100 sccm of hydrogen and 200 sccm of argon for 3 h. The patches also break up via Rayleigh-like instability to form patterns with a greater number of particles than in the initial patch. For patches of identical shape and size, the number of particles inside the resulting dewetted pattern is smallest when the edges are initially normal to the <001> or <110> directions, indicating the relatively greater stability against Rayleigh-like instability along these directions. The edges normal to the <001> and <110> directions in Pd(110) films are crystallographically confined to expose {111} and {100} facets, respectively. As seen in the left-most panels of Fig. 2a,b, in the patterns formed by dewetting of patches whose edges are initially aligned normal to the <001> or <110> directions, fewer particles are formed normal to the <001> directions than normal to the <110> directions, indicating the longer critical wavelength for Rayleigh-like instability of lines with <001> edge normals^13^. This suggests that {111} facets are probably more prominent than {100} facets in the Pd films under the annealing conditions described above.

### The effects of initial film thickness on feature sizes of dewetted patterns

**Figure 3:**
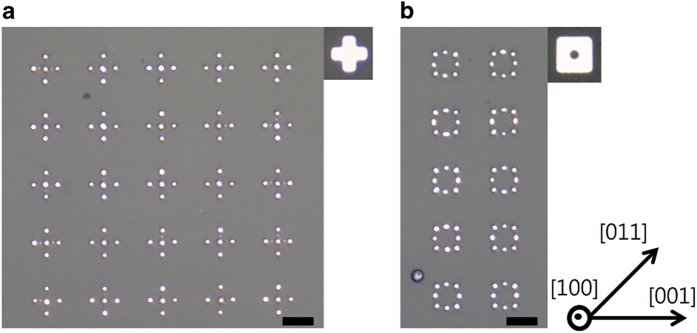
Dewetting of patches patterned from a 30 nm-thick Pd(100) film. (**a**) OM image of patterns produced via dewetting of cross patches. (**b**) OM image of patterns produced via dewetting of square patches with an internal circular hole. The initial patch is shown in the upper-right corner of the corresponding panel. The orientation of the film is indicated in the bottom-right corner of the figure. The scale bars represent 5 μm.

The critical wavelength for Rayleigh-like instability is linearly proportional to the cylinder diameter. Thus, for dewetted patterns produced via Rayleigh-like instability, the size of the features can be reduced by using thinner films. Figure 3 shows the patterns formed during dewetting of patches patterned from a 30 nm-thick Pd(100) film. This film was annealed at 900 °C under 100 sccm of hydrogen and 200 sccm of argon for 30 min. In comparing with the results of Figs. 1,3, it is seen that the dewetted patterns obtained from the 30 nm-thick and 75 nm-thick films differ clearly in scale with smaller and more closely-spaced particles formed from the thinner film. For example, in the dewetting of cross patches with arm widths of 2.5 μm, 3.5 μm, and 4.5 μm, the minimum arm width for the formation of five or more particles is 2.5 μm for the 30 nm-thick films (see Fig. 3a) but 3.5 μm for the 75 nm-thick films. In the smallest patterns that consist of five particles, the average size of the particles located in their arms is approximately 708 nm and 1133 nm in the 30 nm-thick and 75 nm-thick films, respectively. Square patches with an external edge length of 6.5 μm and an internal hole diameter of 1.5 μm evolve into eight particles of average size 800 nm in the 30 nm-thick films (see Fig. 3b) but only two to four particles are formed with larger sizes and interspacings in the 75 nm-thick films.

### Fabrication of hierarchical patterns via templated-dewetting

**Figure 4:**
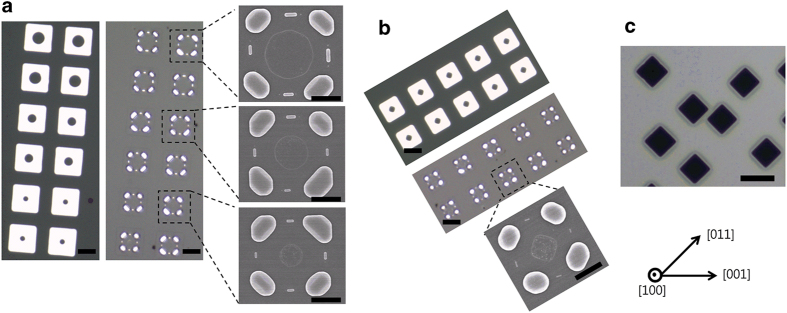
Arrays of hierarchical particle patterns produced from a 75 nm-thick Pd(100) film via dewetting of square patches with an internal circular hole and an external edge length of (**a**) 8.5 μm and (**b**) 6.5 μm, with on either side, OM images of the initial patches and higher-magnification SEM images of the dewetted patterns. (**c**) OM image of natural holes formed in an initially uniform 75 nm-thick Pd(100) film. The orientation of the film is indicated in the bottom-right corner of the figure. The scale bars in the OM images represent 5 μm; the scale bars in the magnified SEM images represent 2 μm.

Arrays of particles of different sizes can also be produced via dewetting of patches patterned from a film without varying the initial thickness. Figure 4 shows such arrays produced via dewetting of square patches with an internal circular hole patterned from a 75 nm-thick Pd(100) film. This film was annealed at 900 °C under 5 sccm of hydrogen and 200 sccm of argon for 1 h. The initial patches shown in Fig. 4a,b were misoriented from the <001> directions by 5° and 30°, respectively. The particle arrays shown in Fig. 4 have hierarchical structures that consist of four relatively large primary particles at the corners and four substantially smaller satellite particles in-between. For the patterns shown in Fig. 4a, the average size of the satellite particles increases from 75 nm to 148 nm and 245 nm as the initial diameter of the internal hole increases from 1.5 μm to 2.5 μm and 3.4 μm, respectively (as measured along the shorter axes of these slightly elongated particles). This can be understood in terms of the evolution of circular holes of different initial diameters. As seen in Fig. 4c, the holes naturally formed during the dewetting of non-patterned Pd(100) films have an in-plane faceted shape with edges normal to the <011> directions. Thompson and I have shown by measuring the retraction rate of pre-patterned long edges of different in-plane orientations that the holes formed naturally in single-crystal films are bound by edges of the lowest retraction rate. However, the rate anisotropy is not sufficiently high that pre-patterned circular holes grow to be bound only by the slowest edges^14^. During the growth of circular holes in patches, along with those normal to the <011> directions, edges normal to the <001> directions also appear and increase in length with the initial diameter of the circular hole. Thus, the patches with a larger internal circular hole evolve during dewetting to form longer lines between the pearled regions at the four convex corners. Indeed, the mean separation distance of the primary particles is greater for the patterns with a larger-diameter hole. This leads to a greater amount of material between the pearled regions separated by Rayleigh-like instability, resulting in larger satellite particles, as seen in Fig. 4a. The satellite particles are smaller on average in Fig. 4b than in Fig. 4a because the initial patches are smaller for the former. It should be noted that these hierarchical patterns of particles were reproduced across the surface of the wafer via dewetting of patches of the same initial shape and in-plane orientation as the ones shown in Fig. 4.

### The effects of film material on pattern formation during templated-dewetting

**Figure 5:**
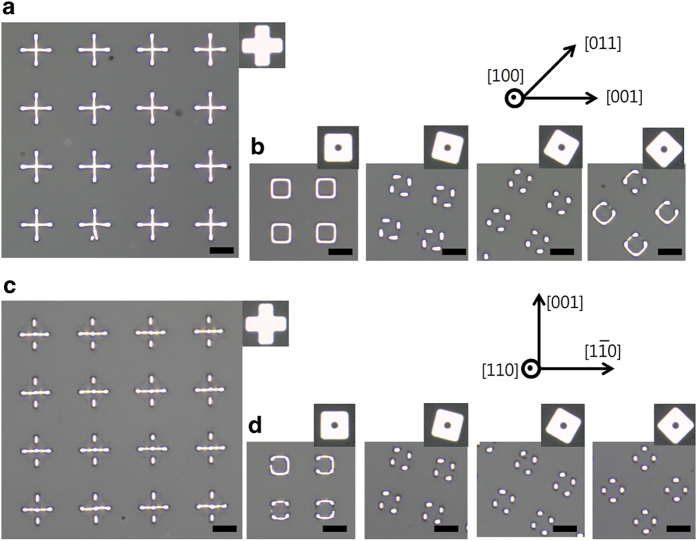
Dewetting of patches patterned from (**a**, **b**) a 75 nm-thick Ni(100) film and (**c**, **d**) a 75 nm-thick Ni(110) film. (**a**, **c**) Patterns produced via dewetting of cross patches. (**b**, **d**) Patterns produced via dewetting of square patches with an internal circular hole. The initial patch is shown in the upper-right corner of the corresponding panel. The orientations of the films are indicated in the upper-right corner and middle-right of the figure. The scale bars represent 5 μm.

Dewetting processes are strongly dependent on the surface energy anisotropy of the film material. Hence, identical patches can dewet into considerably different patterns in films of different materials. Fig. 5a,b shows the results obtained for the dewetting of patches patterned from a 75 nm-thick Ni(100) film. As seen in Fig. 5a, cross patches with arm widths of 3.5 μm evolve into narrower crosses via edge retraction when annealed at 900 °C for 1 h under 5 sccm of hydrogen and 200 sccm of argon. Given that the same patches dewet to form five isolated particles in 75 nm-thick Pd(100) films under the same annealing conditions (see Fig. 1a), this result suggests that the lines or edges normal to the <001> directions are more stable against Rayleigh-like instability in Ni(100) than in Pd(100) films. In the 75 nm-thick Ni(100) films, the results for the dewetting of square patches with an internal hole (see Fig. 5b) show that lines normal to the <001> directions are actually more stable against Rayleigh-like instability than lines aligned normal to other directions. In the Pd(100) films annealed under the same conditions, the most stable lines are normal to the <011> directions, as shown in Fig. 1b. The difference in the in-plane orientation of the most stable line can be understood in terms of the surface energy anisotropy of Ni and Pd. As mentioned earlier, thickening edges of <011> in-plane normals are crystallographically constrained to expose {111} facets in Pd(100) and Ni(100) films. It is known that gamma plots of clean face-centered-cubic metals show the deepest cusps at {111} orientations whose depth is, however, reduced by oxygen adsorbates while adsorption deepens cusps at other orientations^12^,^15^,^16^,^17^. This influence of oxygen adsorption on the surface energy anisotropy was previously characterized by Thompson and I in analyzing the dependence of rim faceting in single-crystal Ni films on the flow rate of the reducing gas. The oxygen adsorbates were removed at a sufficiently high flow rate of reducing gas, leading to the clear appearance of {111} facets. As the flow rate was decreased to increase oxygen adsorption during annealing, {hk0} facets appeared while the {111} facets became less clear^14^. The extent of oxygen adsorption can be different for films of different materials in the same annealing ambient because the driving force for the reduction of the surface oxide is dependent on the film material. Given the smaller driving force for the reduction of nickel oxide in comparison with that for palladium oxide^18^, oxygen adsorption during annealing is likely greater on Ni than on Pd films, causing more prominent faceting at {hk0} orientations and stabilizing the lines with <001> edge normals against Rayleigh-like instability. The patterns in Fig. 5c,d were obtained by dewetting of patches patterned from a 75 nm-thick Ni(110) film. These patches were annealed under the same condition as the ones used for the patches shown in Fig. 2. As seen in the figure, lines normal to the <001> directions show greater stability than ones normal to other directions. This implies that oxygen adsorbates on the Ni(110) film were removed during annealing at the high flow rate (100 sccm) of hydrogen, so that clear {111} faceting occurs and enhances the stability of these lines.

### Fabrication of highly complex patterns via templated-dewetting

**Figure 6:**
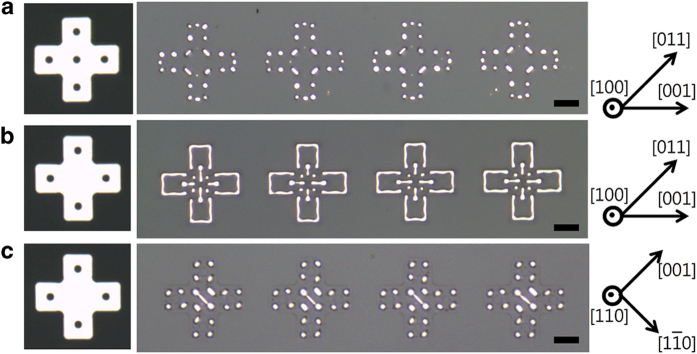
OM images of complex patterns produced via dewetting of cross patches with internal circular holes. The patches were patterned from (**a**) a 30 nm-thick Pd(100) film, (**b**) a 50 nm-thick Ni(100) film, and (**c**) a 75 nm-thick Ni(110) film. The initial patch and the orientation of the films are respectively shown on the left and right of each panel. The scale bars represent 5 μm.

Figure 6 shows highly complex patterns formed via dewetting of cross patches with internal circular holes. In general, shape characteristics of these patterns were reproduced in arrays of patches as seen in the figure. The patterns in Fig. 6a were obtained by dewetting of cross patches with internal circular holes located in the center of their bodies and arms. These patches were patterned from a 30 nm-thick Pd(100) film and annealed at 900°C under 100 sccm of hydrogen and 200 sccm of argon for 30 min. The retraction of the external edges and the growth of the internal holes lead to the formation of lines that subsequently decay into particle arrays via Rayleigh-like instability. As shown in Figs. 1, 2, 3, 4, 5, the stability against Rayleigh-like instability is dependent on the material, thickness, and orientation of the patches. Hence, varying these parameters can lead to the formation of regular particle- and line-based patterns of various shapes during dewetting. Fig. 6b shows results of dewetting of cross patches with internal circular holes located in the center of their arms. These patches were patterned from a 50 nm-thick Ni(100) film and annealed at 900°C under 5 sccm of hydrogen and 200 sccm of argon for 30 min. The external lines of these patterns are normal to the <001> -directions and are therefore resistant to Rayleigh-like instability under this annealing condition. The shape of the central pattern can be explained by the unstable growth of internal holes towards the center. Holes in 50 nm-thick Ni(100) films grow into square shapes with <001> edges that subsequently become unstable during dewetting under the annealing condition described above. As shown in previous studies, the retraction of unstable edges lags near their center, leading to the formation of lines therein^19^. The growth of the four internal holes results in the formation of the lines seen in the center of the patterns of Fig. 6b. The four particles near the center of the pattern are formed through the break-up of non- <001> lines near the retracting concave corners of the crosses. The patterns in Fig. 6c were obtained by dewetting of patches patterned from a 75 nm-thick Ni(110) film. These patches were annealed at 900°C under 100 sccm of hydrogen and 200 sccm of argon for 3 h. Due to large misalignments of the initial edges from the <001> directions, the lines formed during dewetting are not normal to the <001> directions, other than those in the center of the patterns. As shown in Fig. 5c,d, for 75 nm-thick Ni(110) films annealed under these conditions, lines normal to the <001> directions are the most stable against Rayleigh-like instability. In Fig. 6c, therefore, the lines in the center of the patterns remain, but those located elsewhere decay.

## Conclusion

In summary, this paper presents results of dewetting of patches patterned from single-crystal palladium and nickel films of varying thicknesses and orientations. During dewetting, the patches evolve into regular particle- or line-based patterns with smaller feature sizes and increased complexity. The geometric characteristics of each pattern strongly depend on the width, thickness, orientation, and material of the patches. The particle size and interspacing in multi-particle patterns decrease with decreasing the width and thickness of the patches, and the material and orientation of the patches define particular directions along which the stability of the lines is enhanced. The dewetting of patches of particular shapes and in-plane orientations can lead to the formation of regular hierarchical particle patterns. The satellite particles therein are substantially smaller than those in other patterns. The shape and size characteristics of the patterns obtained in this study are explained in terms of the influence of the patch geometry and the surface energy anisotropy of the film material on the development and growth of Rayleigh-like instability. The results of this work demonstrate that dewetting of pre-patterned single-crystal films can be exploited to fabricate wafer-scale arrays of regular patterns with control over the size and shape of their features.

### Outlook

The results presented in this work show that regular patterns can be fabricated with control over their shapes and feature sizes via templated-dewetting of single-crystal films. The smallest features prepared in this way are on the sub-hundred-nanometer scale. As outlined here, however, given their dependence on film thickness, smaller features are probably achievable by dewetting of smaller patches patterned from thinner films than the ones used in this work. The fabrication of smaller patches is in progress by using nanoimprint lithography. The differences found here in the dewetting behaviors of Ni and Pd films are consistent with their surface energy anisotropies, suggesting that the controllable production of regular nanoscale patterns via templated-dewetting is also feasible for other materials of known surface energy anisotropy.

The mechanistic understandings developed in this work can provide predictability for the shape and feature size of patterns produced by templated-dewetting of single-crystal films. Nevertheless, robust 3-dimensional models should be developed to improve the predictability and to realize model-based highly precise pattern formation. Dewetting of pre-patterned single-crystal films has previously been modeled in consideration of surface energy anisotropy, but the modeled pattern geometries are mostly limited to 2-dimensional cross-sections^20^,^21^. The evolution of 3-dimensional anisotropic crystals has been shown to be systematically and reliably predicted via numerical simulation using the facet-velocity polar plot deduced from experimental data^22^,^23^. Given that kinetic and morphological analyses can provide thermodynamic and kinetic data necessary for the model development, I expect that this combined experimental and theoretical approach might also be applicable to predicting the evolution process during templated-dewetting of single-crystal films.

## Methods

The Ni and Pd films used in this study were deposited at 320 °C and 400 °C, respectively, using electron beam evaporation on epi-polished single-crystal MgO(100) and MgO(110) substrates purchased from Crystec. The MgO substrates were heated and outgassed prior to the depositions. The depositions were carried out at a deposition rate of 0.5 Å/s under pressures that varied between 2 × 10^−6^ Torr and 4 × 10^−6^ Torr. The films were patterned into patches using conventional contact photolithography and inductively coupled plasma (ICP) etching of the films. The ICP etching processes were performed at a working pressure of 1 mTorr under 5 sccm of Cl_2_ and 20 sccm of Ar. To induce dewetting, the pre-patterned films were thermally annealed in a quartz tube furnace at 900 °C under Ar and H_2_ ambient. The temperature inside the quartz tube was calibrated with a K-type thermoprobe, and the flow rates of Ar and H_2_ were controlled using mass-flow controllers. The morphologies of the dewetted patterns were investigated using optical microscopy (OM) and scanning electron microscopy (SEM).

## Additional Information

**How to cite this article**: Ye, J. Fabrication of ordered arrays of micro- and nanoscale features with control over their shape and size via templated solid-state dewetting. *Sci. Rep*. **5**, 9823; doi: 10.1038/srep09823 (2015).

## References

[b1] Jiran E. & Thompson C. V. Capillary instabilities in thin films. J. Electron. Mater. 19, 1153 (1990).

[b2] Srolovitz D. J. & Safran S. A. Capillary instabilities in thin films. I. Energetics J. Appl. Phys. 60, 247 (1986).

[b3] Kan W. & Wong H. Fingering instability of a retracting solid film edge. J. Appl. Phys. 97, 043515 (2005).

[b4] Wong H., Voorhees P. W., Miksis M. J. & Davis S. H. Periodic mass shedding of a retracting solid film step. Acta Mater. 48, 1719–1728 (2000).

[b5] Ye J. & Thompson C. V. Regular pattern formation through the retraction and pinch-off of edges during solid-state dewetting of patterned single crystal films. Phys. Rev. B. 82, 193408 (2010).

[b6] Nichols, F. A. & Mullins, W. W. Surface-(interface-) and volume-diffusion contributions to morphological changes driven by capillarity. Trans. Metall. Soc. AIME . 233, 1840 (1965).

[b7] Atwater H. A. & Polman A. Plasmonics for improved photovoltaic devices. Nat. Mater. 9, 205–213 (2010). 20168344 10.1038/nmat2629

[b8] Baumann N., Mutoro E. & Janek J. Porous model type electrodes by induced dewetting of thin Pt films on YSZ substrates. *Solid State Ion*. 181, 7–15 (2010).

[b9] Chhowalla M. *et al.* Growth process conditions of vertically aligned carbon nanotubes using plasma enhanced chemical vapor deposition. J. Appl. Phys. 90, 5308–5317 (2001).

[b10] Giermann A. L. & Thompson C. V. Solid-state dewetting for ordered arrays of crystallographically oriented metal particles. Appl. Phys. Lett. 86, 121903 (2005).

[b11] Ye J. & Thompson C. V. Templated Solid-State Dewetting to Controllably Produce Complex Patterns. Adv. Mater. 23, 1567–1571 (2011). 21449063 10.1002/adma.201004095

[b12] Foiles S. M., Baskes M. I. & S. D. M. Embedded-atom-method functions for the fcc metals Cu, Ag, Au, Ni, Pd, Pt, and their alloys. Phys. Rev. B. 33, 7983 (1986). 10.1103/physrevb.33.7983 9938188

[b13] Cahn J.W. Stability of rods with anisotropic surface free energy. *Scripta Metall. Mater*. 13, 1069 (1979).

[b14] Ye J. P. & Thompson C. V. Anisotropic edge retraction and hole growth during solid-state dewetting of single crystal nickel thin films. Acta Mater. 59, 582–589 (2011).

[b15] Blakely J. M. & Mykura H. Anisotropic edge retraction and hole growth during solid-state dewetting of single crystal nickel thin films. *Acta Metall. Mater*. 9, 595 (1961).

[b16] Mykura H. The variation of the surface tension of nickel with crystallographic orientation. *Acta Metall. Mater*. 9, 570 (1961).

[b17] Seriani N. & Mittendorfer F. Platinum-group and noble metals under oxidizing conditions. J. Phys.: Condens. Matter. 20, 184023 (2008).

[b18] Gaskell D. R. Introduction to the Thermodynamics of Materials . Taylor & Francis: New York, 1995).

[b19] Ye J. P. & Thompson C. V. Mechanisms of complex morphological evolution during solid-state dewetting of single-crystal nickel thin films. Appl. Phys. Lett. 97, 071904 (2010).

[b20] Zucker R.V., Kim G. H., Carter W. C. & Thompson C. V., A model for solid-state dewetting of a fully-faceted thin film. Comptes Rendus Physique 14, 564 (2013).

[b21] Dornel E., Barbe J. C., de Crecy F., Lacolle G. & Eymery J. Surface diffusion dewetting of thin solid films: Numerical method and application to Si/SiO2. Physical Review B. 73, 115427 (2006).

[b22] Du D. X., Srolovitz D. J., Coltrin M. E. & Mitchell C. C. Systematic prediction of kinetically limited crystal growth morphologies. Physical Review Letters 95, 155503 (2005). 16241736 10.1103/PhysRevLett.95.155503

[b23] Sun Q. *et al.* Understanding nonpolar GaN growth through kinetic Wulff plots. Journal of Applied Physics 104, 093523 (2008).

